# Supplementary material for: Content-rich biological network constructed by mining PubMed abstracts
Source: BMC Bioinformatics. 2004 Oct 8;5:147. doi: 10.1186/1471-2105-5-147 (PMC528731; doi:10.1186/1471-2105-5-147)
Supplement: Additional File 5 — The original Chilibot query results of the term "long-term potentiation (LTP)" and 22 other terms, limiting the latest references analyzed to the years 1990, 1995, 2000, and 2004. [file 1471-2105-5-147-S5.bz2 › chilibotAdditionalFile5/ltp2004/html/TRKB_ACTIN.html]

 


 **TRKB** and **ACTIN** 
  
Found 6 abstracts in PubMed,  **6 abstracts were retrieved and analyzed**.  


---

 Search Google  |
 PDF files only 
|  EDU domain only 

---

**Interactive relationship** (e.g. stimulation, inhibition, etc)

- No  **TrkB**  positive cells exhibited acetylcholine receptor clusters or were positive for alpha smooth muscle  **actin** .  Ref: 11083466 Histochem Cell Biol, 2000
- To demonstrate spatial relationships of  **TrkB**  immunoreactive cells to striated and smooth muscle fibers we combined  **TrkB**  immunocytochemistry with fluorochrome tagged alpha bungarotoxin for labeling of nicotinic acetylcholine receptors, and alpha smooth muscle  **actin**  for labeling of smooth muscle cells.  Ref: 11083466 Histochem Cell Biol, 2000

**Parallel relationship** (e.g. studied together, co-existance, homology, etc.)

- Two PCR protocols are described where single cell cDNA was analysed for  **TrkB**  and GAPDH or  **TrkB** , TrkC, Ret, Met, ErbB2 and Beta  **actin**  by multiplex PCR.  Ref: 12431706 Brain Res Brain Res Protoc, 2002
